# Supplementary material for: Genome-wide association study of pulpal and apical diseases
Source: Nat Commun. 2025 Jul 23;16:6774. doi: 10.1038/s41467-025-61721-1 (PMC12287303; doi:10.1038/s41467-025-61721-1)
Supplement: Supplementary file 1 — Supplementary Information [file 41467_2025_61721_MOESM1_ESM.pdf]

Supplementary Information

Genome-wide association study of pulpal and apical diseases

Aino Salminen[1], Kati Hyvärinen[2], Jarmo Ritari[2], Jussi M. Leppilahti[3,4,5], Ulla Palotie[1], Ville Vuollo[3,5], Oleg Kambur[6,7,8,9], FinnGen, Estonian Biobank Research Team, Kadri Reis[10], Anu Reigo[10], Priit Palta[10], Markus Perola[6], Juha Sinisalo[11], Aki S. Havulinna[6,12,13], Päivi Mäntylä[7], Ulvi Kahraman Gürsoy[14], A. Liisa Suominen[7,15], David P. Rice[1], Vuokko Anttonen[16], Pekka Nieminen[1], Pirkko J. Pussinen[1,7]

[1] Oral and Maxillofacial Diseases, University of Helsinki and Helsinki University Hospital, Helsinki, Finland
[2] Finnish Red Cross Blood Service, Research and Development, Helsinki, Finland
[3] Research Unit of Oral Health Sciences, Faculty of Medicine, University of Oulu, Oulu, Finland
[4] Department of Oral and Maxillofacial Surgery, Oulu University Hospital, Oulu, Finland
[5] Medical Research Center Oulu, Oulu University Hospital and University of Oulu, Oulu, Finland
[6] Finnish Institute for Health and Welfare, Helsinki, Finland
[7] School of Medicine, Institute of Dentistry, University of Eastern Finland, Kuopio, Finland
[8] Department of Internal Medicine, University of Turku, Turku, Finland
[9] Department of Pharmacology, Faculty of Medicine, University of Helsinki, Helsinki, Finland
[10] Estonian Genome Centre, Institute of Genomics, University of Tartu, Tartu, Estonia
[11] Heart and Lung Center, Helsinki University Hospital and University of Helsinki, Helsinki, Finland
[12] Institute for Molecular Medicine Finland (FIMM), HiLIFE, University of Helsinki, Helsinki, Finland
[13] Department of Computing, Faculty of Technology, University of Turku, Turku, Finland
[14] Department of Periodontology, Institute of Dentistry, University of Turku, Turku, Finland
[15] Oral and Maxillofacial Teaching Unit, Kuopio University Hospital, Kuopio, Finland
[16] Research Unit of Population Health, University of Oulu, Oulu, Finland

Supplementary Information

Contents

Supplementary Figure 1. Numbers of individuals with different diagnosis codes within the K04 category of ICD-10 system.

Supplementary Figure 2. Locus zooms of the GWAS-significant loci for the phenotype Pulpal and apical diseases.

Supplementary Figure 3. Locus zooms of the GWAS-significant loci for the phenotype Pulpitis.

Supplementary Figure 4. Locus zooms of the GWAS-significant loci for the phenotype Necrosis of pulp or apical periodontitis.

Supplementary Figure 5. Observed heritability estimates (H^2) of the FinnGen phenotypes.

Supplementary Table 1. Replication of the genetic associations found in the discovery analysis.

Supplementary Table 2. Replicated SNPs from an earlier GWAS on apical periodontitis.

Supplementary Table 3. *In silico* analyses of the lead SNPs for endodontic phenotypes.

Supplementary Table 4. Gene Ontology of the protein-coding gene families for the endodontic phenotypes.

Supplementary Table 5. List of phenotypes included in the endpoint autoimmune diseases.

Supplementary Table 6. Replication of the associations between endodontic phenotypes and HLA alleles in FinnGen-replication.

Supplementary Table 7. Replication of the associations of KIR gene contents with endodontic phenotypes in FinnGen-replication.

Supplementary Table 8. Significant associations of the top SNPs with other phenotypes in FinnGen.

Supplementary Table 9. Observed heritability estimates of the FinnGen phenotypes.

Supplementary Table 10. Genetic correlations between endodontic phenotypes and selected FinnGen phenotypes.

Figures

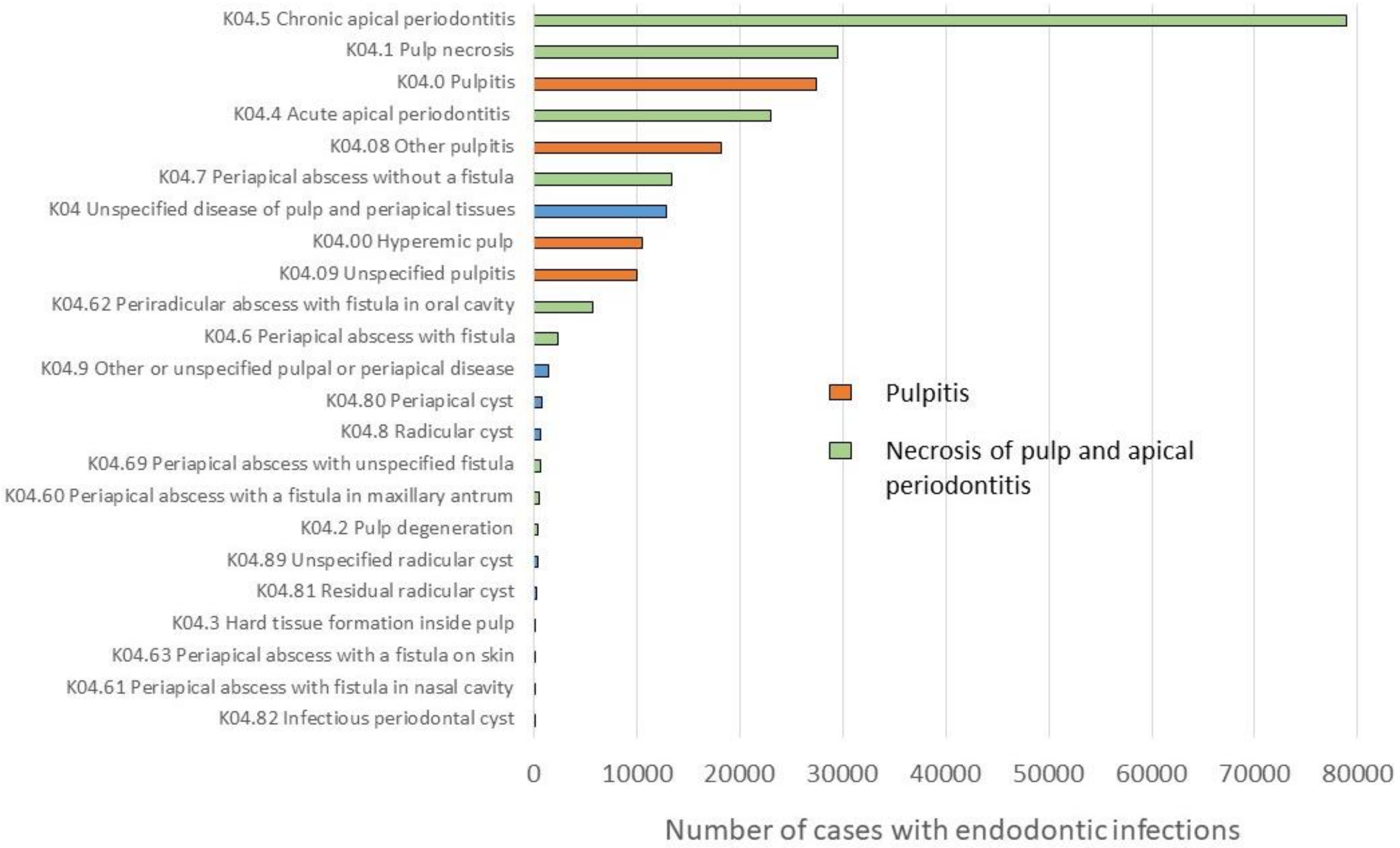

Supplementary Figure 1. Numbers of individuals with different diagnosis codes within the K04 category of ICD-10 system. Phenotype and covariate data was available from 485,230 individuals in FinnGen Release 12 for the discovery analyses. Number of cases with any of the ICD-10 codes for pulpal or apical diseases was 132,124. Two sub-phenotypes were designed: i) Pulpitis with ICD-10 codes K04.1, K04.08, K04.00, and K04.09; and ii) Necrosis of pulp or apical periodontitis with ICD-10 diagnosis codes K04.1, K04.2, K04.3, K04.4, K04.5, K04.6, K04.60, K04.61, K04.62, K04.63, K04.69, and K04.7

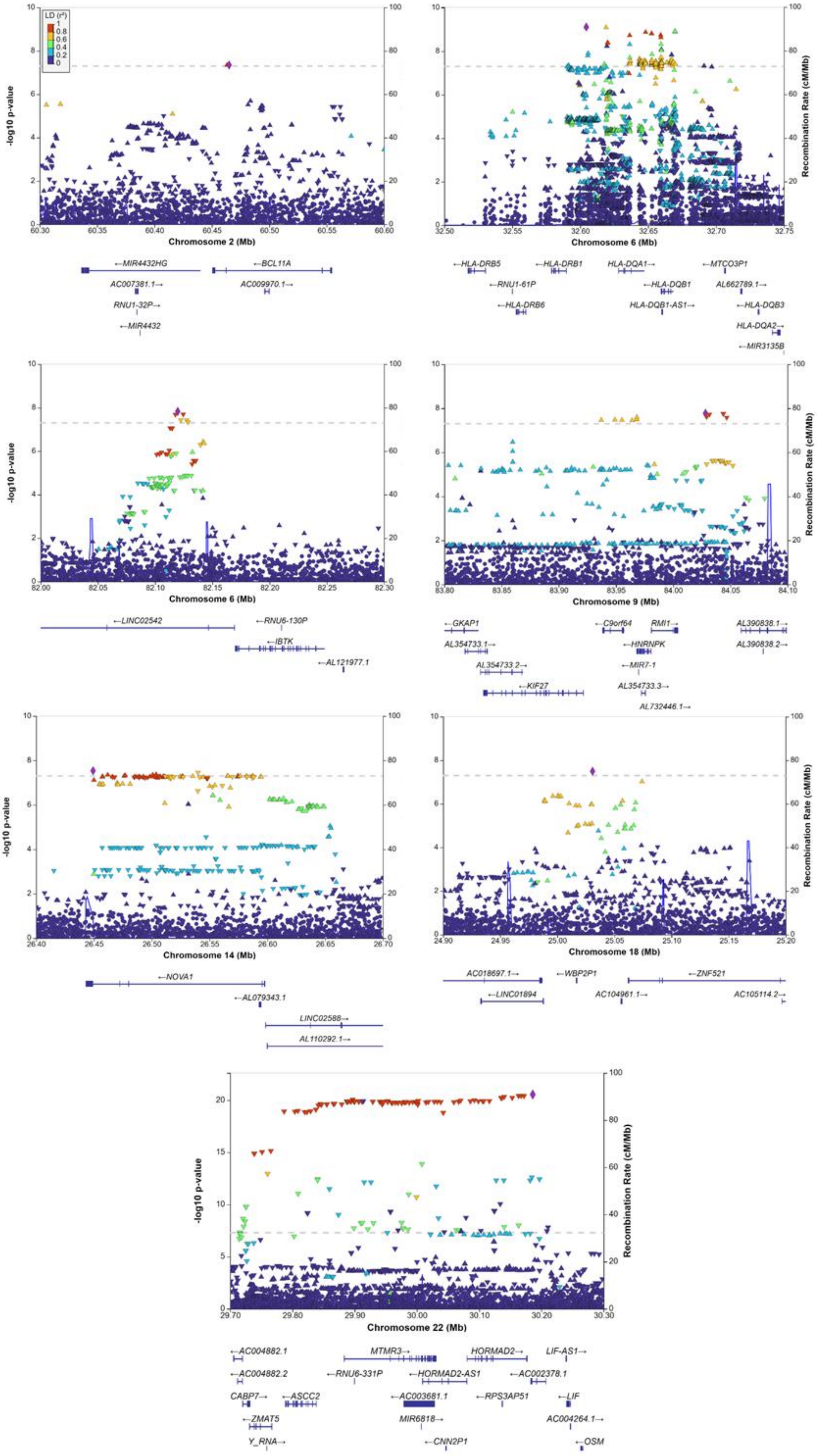

-log10 p-value
Recombination Rate (cM/Mb)
LD (r²)
Chromosome 2 (Mb)
←MIR4432HG
←BCL11A
AC007381.1→
AC009970.1→
RNU1-32P→
←MIR4432
Chromosome 6 (Mb)
←HLA-DRB5
←HLA-DRB1
HLA-DQA1→
←MTCO3P1
←RNU1-61P
←HLA-DQB1
AL662789.1→
←HLA-DRB6
HLA-DQB1-AS1→
←HLA-DQB3
HLA-DQA2→
←MIR3135B
Chromosome 6 (Mb)
←LINC02542
←RNU6-130P
←IBTK
←AL121977.1
Chromosome 9 (Mb)
←GKAP1
←C9orf64
RMI1→
AL390838.1→
AL354733.1→
←HNRNPK
AL390838.2→
AL354733.2→
←MIR7-1
←KIF27
AL354733.3→
AL732446.1→
Chromosome 14 (Mb)
←NOVA1
←AL079343.1
LINC02588→
AL110292.1→
Chromosome 18 (Mb)
AC018697.1→
←WBP2P1
←ZNF521
←LINC01894
AC104961.1→
AC105114.2→
Chromosome 22 (Mb)
←AC004882.1
MTMR3→
HORMAD2→
LIF-AS1→
←AC004882.2
←RNU6-331P
←HORMAD2-AS1
←AC002378.1
CABP7→
←ASCC2
←AC003681.1
←RPS3AP51
←LIF
←ZMAT5
MIR6818→
AC004264.1→
Y_RNA→
←CNN2P1
←OSM

Supplementary Figure 2. Locus zooms of the GWAS-significant loci for the phenotype Pulpal and apical diseases. The analysis included 132,124 cases and 353,106 controls. Associations between each SNP and phenotypes were analyzed with an additive model. For each SNP, linkage disequilibrium (LD) with the lead SNP is indicated by colour coding. The dashed line indicates the threshold for genome-wide significance $p < 5*10^{-8}$. A) Chr 2, lead SNP rs143351662; B) Chr 6, lead SNP rs9270911; C) Chr 6, lead SNP rs9443960; D) Chr 9, lead SNP rs10046912; E) Chr 14, lead SNP rs1245193; F) Chr 18, lead SNP rs8088342; G) Chr 22, lead SNP rs9614155.

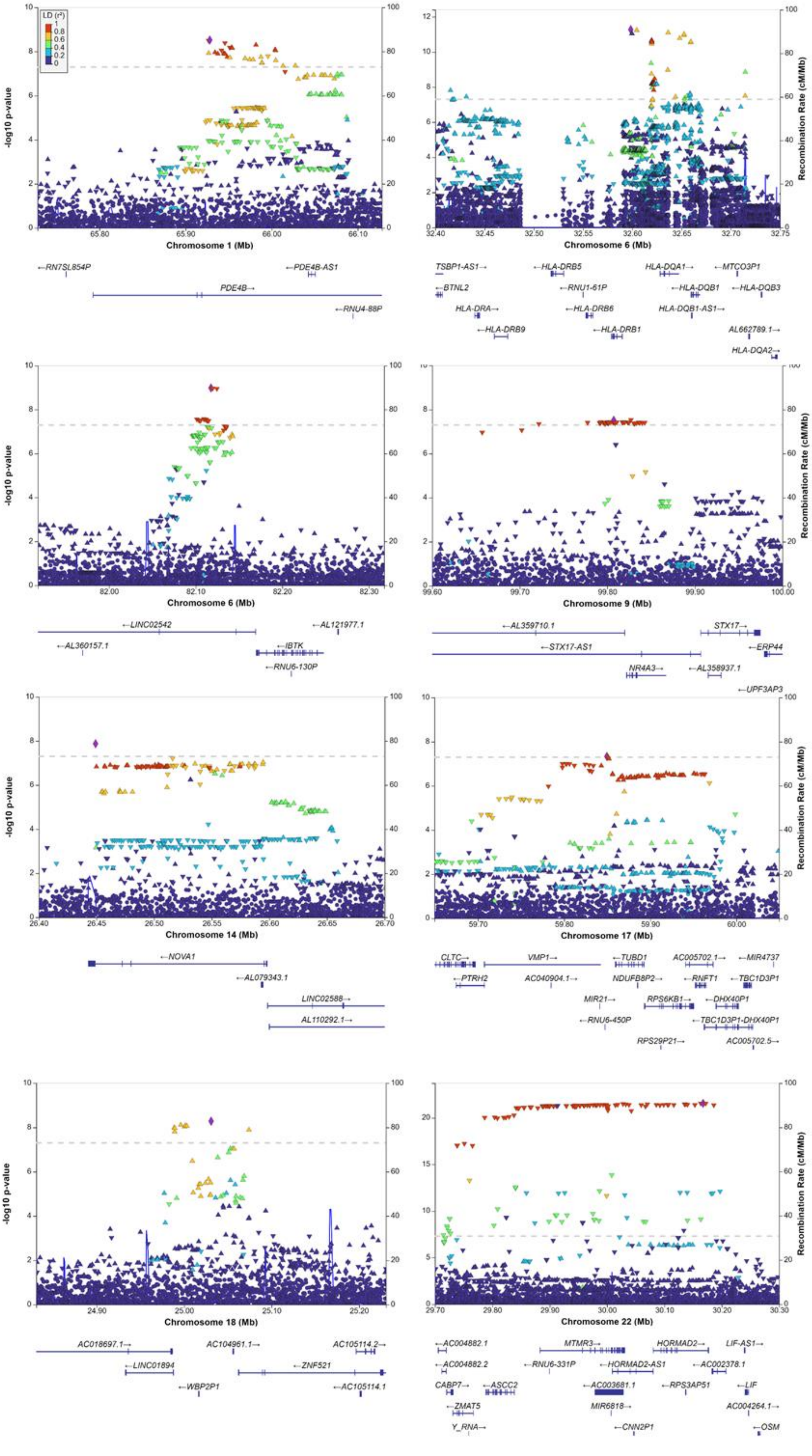

LD (r²)
-log10 p-value
Recombination Rate (cM/Mb)
Chromosome 1 (Mb)
←RN7SL854P
←PDE4B-AS1
PDE4B→
←RNU4-88P
Chromosome 6 (Mb)
TSBP1-AS1→
←BTNL2
HLA-DRA→
←HLA-DRB9
←HLA-DRB5
←RNU1-61P
←HLA-DRB6
←HLA-DRB1
HLA-DQA1→
←HLA-DQB1
HLA-DQB1-AS1→
←MTCO3P1
←HLA-DQB3
AL662789.1→
HLA-DQA2→
Chromosome 6 (Mb)
←LINC02542
←AL360157.1
←AL121977.1
←IBTK
←RNU6-130P
Chromosome 9 (Mb)
←AL359710.1
←STX17-AS1
NR4A3→
STX17→
←ERP44
←AL358937.1
←UPF3AP3
Chromosome 14 (Mb)
←NOVA1
←AL079343.1
LINC02588→
AL110292.1→
Chromosome 17 (Mb)
CLTC→
←PTRH2
VMP1→
AC040904.1→
MIR21→
←RNU6-450P
←TUBD1
NDUFB8P2→
RPS6KB1→
RPS29P21→
AC005702.1→
←RNFT1
←DHX40P1
←TBC1D3P1-DHX40P1
←MIR4737
←TBC1D3P1
AC005702.5→
Chromosome 18 (Mb)
AC018697.1→
←LINC01894
AC104961.1→
←WBP2P1
←ZNF521
AC105114.2→
←AC105114.1
Chromosome 22 (Mb)
←AC004882.1
←AC004882.2
CABP7→
←ZMAT5
Y_RNA→
←ASCC2
←RNU6-331P
MTMR3→
←HORMAD2-AS1
←AC003681.1
MIR6818→
←CNN2P1
HORMAD2→
←AC002378.1
←RPS3AP51
LIF-AS1→
←LIF
AC004264.1→
←OSM

Supplementary Figure 3. Locus zooms of the GWAS-significant loci for the phenotype Pulpitis. The analysis included 48,120 cases and 353,106 controls. Associations between each SNP and phenotypes were analyzed with an additive model. For each SNP, linkage disequilibrium (LD) with the lead SNP is indicated by colour coding. The dashed line indicates the threshold for genome-wide significance $p < 5*10^{-8}$. A) Chr 1, lead SNP rs2997084; B) Chr6, lead SNP rs9270664; C) Chr 6, lead SNP rs9449423; D) Chr 9, lead SNP rs1417738; E) Chr 14, lead SNP rs1245193; F) Chr 17, lead SNP rs1292071; G) Chr 18, lead SNP rs8088342; H) Chr 22, lead SNP rs9614152.

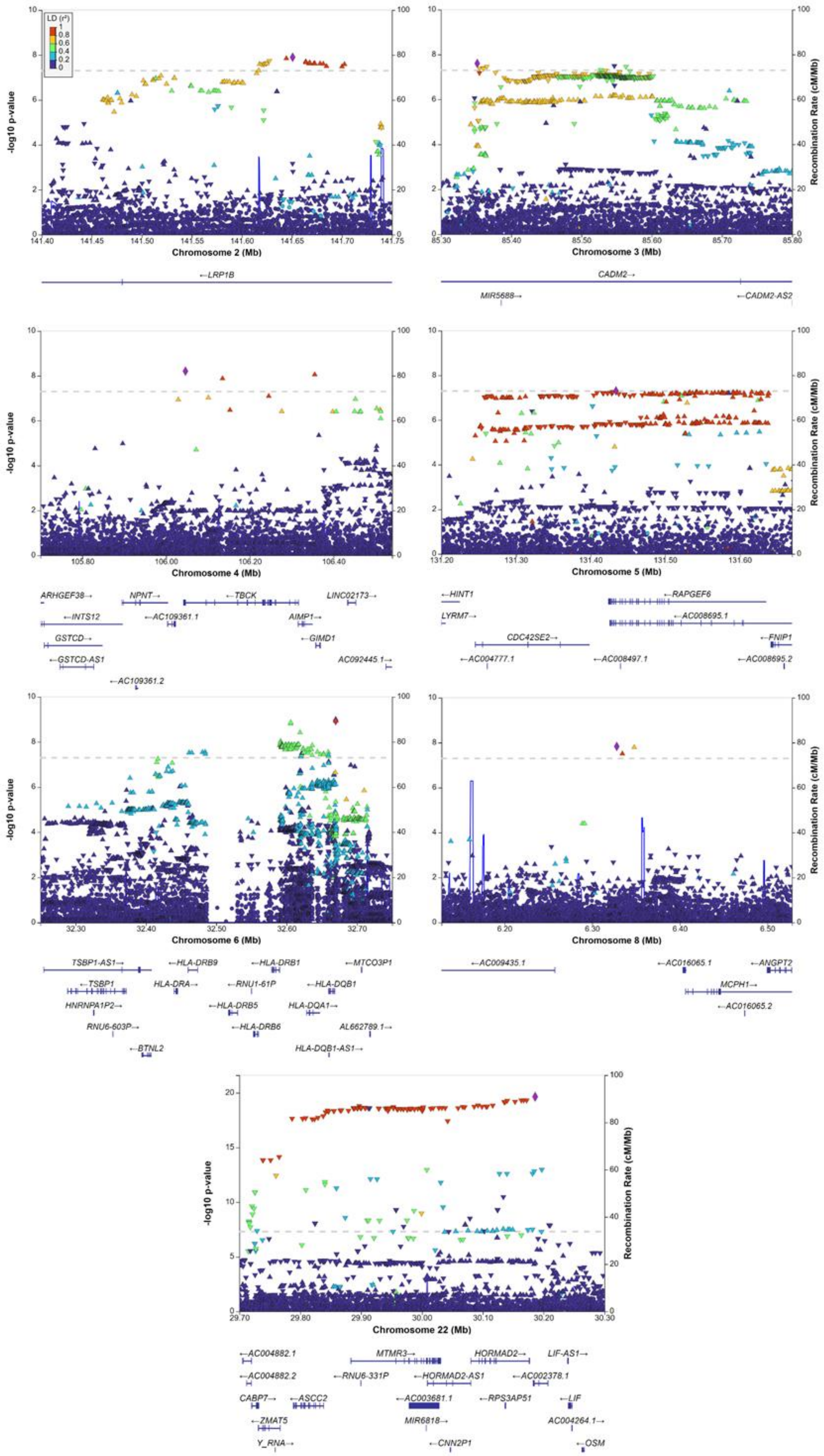

LD (r²)
1
0.8
0.6
0.4
0.2
0
-log10 p-value
Recombination Rate (cM/Mb)
Chromosome 2 (Mb)
←LRP1B
Chromosome 3 (Mb)
CADM2→
MIR5688→
←CADM2-AS2
Chromosome 4 (Mb)
ARHGEF38→
NPNT→
←TBCK
LINC02173→
←INTS12
←AC109361.1
AIMP1→
GSTCD→
←GIMD1
←GSTCD-AS1
AC092445.1→
←AC109361.2
Chromosome 5 (Mb)
←HINT1
←RAPGEF6
LYRM7→
←AC008695.1
CDC42SE2→
←FNIP1
←AC004777.1
←AC008497.1
←AC008695.2
Chromosome 6 (Mb)
TSBP1-AS1→
←HLA-DRB9
←HLA-DRB1
←MTCO3P1
←TSBP1
HLA-DRA→
←RNU1-61P
←HLA-DQB1
HNRNPA1P2→
←HLA-DRB5
HLA-DQA1→
RNU6-603P→
←HLA-DRB6
AL662789.1→
←BTNL2
HLA-DQB1-AS1→
Chromosome 8 (Mb)
←AC009435.1
←AC016065.1
←ANGPT2
MCPH1→
←AC016065.2
Chromosome 22 (Mb)
←AC004882.1
MTMR3→
HORMAD2→
LIF-AS1→
←AC004882.2
←RNU6-331P
←HORMAD2-AS1
←AC002378.1
CABP7→
←ASCC2
←AC003681.1
←RPS3AP51
←LIF
←ZMAT5
MIR6818→
AC004264.1→
Y_RNA→
←CNN2P1
←OSM

Supplementary Figure 4. Locus zooms of the GWAS-significant loci for the phenotype Necrosis of pulp or apical periodontitis. The analysis included 103,832 cases and 353,106 controls. Associations between each SNP and phenotypes were analyzed with an additive model. For each SNP, linkage disequilibrium (LD) with the lead SNP is indicated by colour coding. The dashed line indicates the threshold for genome-wide significance $p < 5*10^{-8}$. A) Chr 2, lead SNP rs36044739; B) Chr 3, lead SNP rs62253088; C) Chr 4, lead SNP rs79409042; D) Chr 5, lead SNP rs6596024; E) Chr 6, lead SNP rs9274719; F) Chr 8, lead SNP rs35858015; G) Chr 22, lead SNP rs9614155.

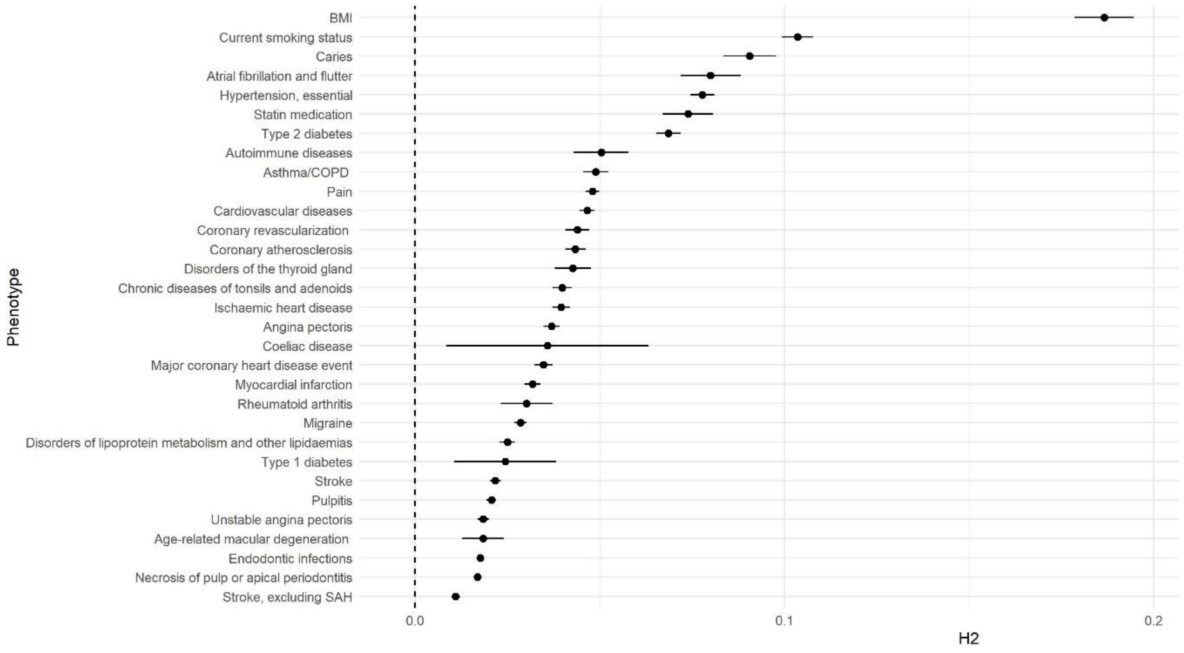

Supplementary Figure 5. Observed heritability estimates (H^2) of the FinnGen phenotypes. For each phenotype, genome-wide single nucleotide polymorphism (SNP)-based heritability (H^2) was estimated using univariate linkage-disequilibrium score regression (LDSR). Dots present heritability estimates and error bars represent standard errors.

Supplementary Table 1. Replication of the genetic associations found in the discovery analysis.

Chr	Lead SNPs from the discovery cohort	Replication 1 FinnGen - replication			Replication 2 EstBB			Replication 3 NFBC	
		Pulpal and apical diseases	Pulpitis	Necrosis of pulp or apical periodontitis	Pulpal and apical diseases	Pulpitis	Necrosis of pulp or apical periodontitis	Deep caries [1] and regular dental pain[2]	Apical lesions [3]
		**Beta (SE), *P*-value**							
**Pulpal and apical diseases**									
2	rs143351662	0.095 (0.086), 0.27	-0.060 (0.123), 0.62	0.111 (0.095), 0.24	0.075 (0.047), 0.11	0.041 (0.081), 0.61	0.078 (0.052), 0.13	-0.031 (0.025), 0.23	0.18 (0.31), 0.57
6	rs9270911	0.049 (0.027), 0.07	0.018 (0.039), 0.64	0.057 (0.030), 0.06	not found	not found	not found	0.007 (0.009), 0.45	**0.24 (0.11), 0.03**
6	rs9443960	0.025 (0.026), 0.34	0.033 (0.038), 0.39	0.001 (0.029), 0.79	-0.014 (0.007), 0.05	-0.001 (0.012), 0.92	**-0.016 (0.008), 0.04**	0.005 (0.009), 0.59	0.097 (0.089), 0.28
9	rs10046912	0.0005 (0.031), 0.99	0.001 (0.044), 0.98	0.008 (0.034), 0.82	-0.001 (0.009), 0.95	-0.019 (0.015), 0.21	0.004 (0.009), 0.71	-0.008 (0.010), 0.44	0.17 (0.11), 0.11
14	rs1245193	**-0.090 (0.035), 0.01**	-0.093 (0.051), 0.07	**-0.083 (0.039), 0.03**	-0.002 (0.009), 0.86	0.015 (0.016), 0.36	-0.005 (0.010), 0.60	**-0.028 (0.011), 0.018**	-0.055 (0.12), 0.64
18	rs8088342	0.014 (0.027), 0.62	**0.088 (0.039), 0.02**	-0.020 (0.030), 0.51	0.006 (0.008), 0.46	0.017 (0.013), 0.18	0.003 (0.008), 0.71	0.0001 (0.009), 0.99	-0.045 (0.089), 0.62
22	rs9614155	**-0.143 (0.037), 0.0001**	**-0.113 (0.052), 0.03**	**-0.178 (0.041), 1.6*10^{-5}**	**-0.037 (0.009), 4.7*10^{-5}**	**-0.062 (0.016), 9.7*10^{-5}**	**-0.039 (0.010), 8.4*10^{-5}**	not found	not found
**Pulpitis**									
1	rs2997084	0.015 (0.027), 0.58	0.027 (0.039), 0.48	-0.001 (0.030), 0.99	-0.0006 (0.007), 0.93	-0.013 (0.012), 0.28	-0.001 (0.008), 0.88	-0.002 (0.009), 0.79	0.081 (0.092), 0.37
6	rs9270664	**0.060 (0.026), 0.02**	0.024 (0.038), 0.53	**0.071 (0.029), 0.02**	not found	not found	not found	0.010 (0.009), 0.26	0.098 (0.11) 0.36
6	rs9449423	0.025 (0.027), 0.34	0.033 (0.038), 0.38	0.001 (0.029), 0.98	**-0.014 (0.007), 0.05**	-0.0009 (0.012), 0.94	**-0.016 (0.008), 0.04**	0.005 (0.009), 0.59	0.097 (0.089) 0.28
9	rs1417738	-0.030 (0.028), 0.29	-0.019 (0.041), 0.64	-0.018 (0.031), 0.57	0.0004 (0.007), 0.95	-0.010 (0.013), 0.44	-0.004 (0.008), 0.63	-0.013 (0.009), 0.16	-0.18 (0.096), 0.07

14	rs1245193	**-0.090 (0.035), 0.01**	-0.093 (0.051), 0.07	**-0.083 (0.039), 0.03**	-0.0016 (0.010), 0.86	0.015 (0.016), 0.36	-0.005 (0.010), 0.60	**-0.028 (0.011), 0.018**	-0.055 (0.12), 0.64
17	rs1292071	0.050 (0.026), 0.06	**0.092 (0.038), 0.02**	0.053 (0.019), 0.07	0.0009 (0.007), 0.90	0.019 (0.012), 0.13	-0.001 (0.008), 0.87	0.012 (0.009), 0.19	-0.074 (0.089), 0.40
18	rs8088342	0.014 (0.027), 0.62	**0.088 (0.039), 0.02**	-0.020 (0.030), 0.51	0.006 (0.008), 0.46	0.017 (0.013), 0.18	0.003 (0.008), 0.71	0.0001 (0.009), 0.99	-0.045 (0.089), 0.62
22	rs9614152	**-0.144 (0.037), 0.0001**	**-0.108 (0.052), 0.04**	**-0.180 (0.041), $1.3*10^{-5}$**	**-0.037 (0.009), $5.0*10^{-5}$**	**-0.061 (0.016), 0.0001**	**-0.039 (0.010), $9.6*10^{-5}$**	-0.004 (0.012), 0.78	0.014 (0.13), 0.91
**Necrosis of pulp or apical periodontitis**									
2	rs36044739	0.076 (0.063), 0.23	0.050 (0.093), 0.59	0.079 (0.069), 0.25	0.016 (0.013), 0.21	0.010 (0.022), 0.65	0.020 (0.014), 0.14	not found	not found
3	rs62253088	-0.046 (0.019), 0.11	-0.057 (0.042), 0.17	-0.046 (0.032), 0.16	0.008 (0.008), 0.32	0.0004 (0.013), 0.98	0.007 (0.009), 0.41	0.003 (0.010), 0.76	-0.048 (0.096) 0.62
4	rs79409042	0.059 (0.068), 0.38	0.031 (0.097), 0.75	0.071 (0.074), 0.34	-0.014 (0.017), 0.39	-0.054 (0.029), 0.06	0.004 (0.018), 0.83	0.017 (0.021), 0.43	-0.23 (0.24) 0.35
5	rs6596024	0.003 (0.028), 0.92	-0.040 (0.347), 0.32	0.003 (0.031), 0.91	-0.012 (0.008), 0.11	**-0.027 (0.013), 0.04**	-0.007 (0.008), 0.41	0.004 (0.009), 0.64	-0.004 (0.094) 0.96
6	rs9274724	0.042 (0.032), 0.19	0.030 (0.046), 0.51	0.031 (0.035), 0.37	not found	not found	not found	not found	not found
8	rs35858015	-0.060 (0.073), 0.41	-0.075 (0.104), 0.47	-0.041 (0.079), 0.61	0.012 (0.015), 0.43	0.024 (0.026), 0.35	0.020 (0.017), 0.24	0.023 (0.026), 0.36	0.008 (0.25) 0.98
22	rs9614155	**-0.143 (0.037), 0.0001**	**-0.113 (0.052), 0.03**	**-0.178 (0.041), $1.6*10^{-5}$**	**-0.037 (0.009), $4.7*10^{-5}$**	**-0.061 (0.016), $9.7*10^{-5}$**	**-0.039 (0.010), $8.4*10^{-5}$**	not found	not found

The additive models were adjusted for age, gender, and 10 genetic principal components. *P*-values < 0.05 were considered significant in the replication analyses. Associations with statistically significant *P*-values and same effect direction as in the discovery analysis are bolded.

[1] ICDAS 5 and 6; [2] dental pain fairly or very often; [3] Apical lesions registered from X-rays.

Supplementary Table 2. Replicated SNPs from an earlier GWAS on apical periodontitis[11].

Chromosome	Position	Alleles	rsID	Nearest gene
1	21672345	C/T	rs12036106	RAP1GAP
2	187805990	C/A	rs13031512	TFPI
4	87938167	A/G	rs72870126	SPP1
4	168601614	TA/T	rs369717575	PALLD
5	109992836	C/T	rs36793	TMEM232
5	116365770	C/T	rs116632893	COMMD10
6	15808827	C/T	rs74291093	DTNBP1
6	106208887	C/A	rs11751513	ATG5
6	131538674	T/C	rs2749929	ARG1
6	167723878	T/C	rs148550758	LINC02487
8	17523673	T/C	rs139604353	SLC7A2
8	24019547	C/G	rs7835237	STC1
10	1260605	G/A	rs868304463	ADARB2
11	69104347	G/C	rs12800372	TPCN2
11	134665535	G/A	rs59613820	LINC02714
12	1554407	G/A	rs886538	FBXL14
13	25384277	A/G	rs1924782	ATP8A2
18	66145400	C/A	rs146374374	CDH7

Supplementary Table 3. ***In silico*** **analyses of the lead SNPs for endodontic phenotypes.**

Chr	rsID	Nearest gene	Type	FIVEx<br>Symbol (tissue); down/up	*cis*-eQTLGen phase I	pQTL
**Pulpal and apical diseases**						
**2**	rs143351662	BCL11A	intron	no	no	no
**6**	rs9270911	HLA-DRB1	intergenic	HLA-DRB1 (multiple); down<br>HLA-DRB5 (multiple); down<br>HLA-DQA1 (multiple); down<br>HLA-DQB1 (multiple); down<br>HLA-DQA2 (multiple); up<br>HLA-DQB2 (multiple); up<br>HLA-DQB1-AS1 (multiple); down	HLA-DQB1<br>HLA-DRB5<br>HLA-DQA1<br>HLA-DQB1-AS1<br>HLA-DQA2<br>HLA-DQB2<br>HLA-DRB1<br>C4B<br>HLA-DRB6<br>C4A<br>CYP21A2<br>AGER<br>XXbac-BPG246D15.8<br>RNF5<br>ATF6B<br>MSH5<br>PPP1R2P1<br>TNXB	CCL19<br>CDSN<br>HLA-DRA<br>MICB<br>TNXB<br>HERC5<br>RACGAP1<br>SH3GL3<br>METTL2B<br>MICA
**6**	rs9443960	LINC02542	intergenic	no	no	no
**9**	rs10046912	RMI1	intergenic	RMI1 (Cell Culture); up<br>RMI1 (Muscle); up<br>GKAP1 (Immune); up	RMI1<br>GKAP1<br>KIF27<br>IDNK	no
**14**	rs1245193	NOVA1	intron	no	no	no
**18**	rs8088342	WBP2P1	intergenic	no	no	no

**22**	rs9614155	HORMAD2	intron	AC003681.1 (MTMR3) (multiple in immune, adipose, stomach, skin, adrenal gland, thyroid, reproductive, esophagus, brain, blood vessel); up	MTMR3<br>ASCC2<br>SF3A1<br>THOC5<br>GATSL3<br>NEFH<br>DUSP18	no
**Pulpitis**						
**1**	rs2997084	PDE4B	intron	no	LEPROT<br>LEPR	no
**6**	rs9270664	HLA-DRB1	intergenic	HLA-DRB1 (multiple); down<br>HLA-DRB5 (multiple); down<br>HLA-DQA1 (multiple); down<br>HLA-DQB1 (multiple); down<br>HLA-DQA2 (multiple); up<br>HLA-DQB2 (multiple); up<br>HLA-DQB1-AS1 (multiple); down	no	CDSN<br>HLA-DRA<br>MICB<br>TNXB<br>SH3GL3<br>METTL2B
**6**	rs9449423	LINC02542	intergenic	no	no	no
**9**	rs1417738	NR4A3	intron	no	INVS<br>ERP44	no
**14**	rs1245193	NOVA1	intron	no	no	no
**17**	rs1292071	VMP1	intergenic	RNFT1-DT (brain, tibial nerve, subcutaneous adipose, thyroid, T-cell); up<br>RNFT1 (blood); down<br>VMP1 (naïve macrophage); up	RNFT1<br>HEATR6<br>RP11-3K24.1<br>TUBD1<br>YPEL2	no
**18**	rs8088342	WBP2P1	intergenic	no	no	no

**22**	rs9614152	HORMAD2	intron	AC003681.1 (MTMR3) (multiple in immune, adipose, stomach, skin, adrenal gland, thyroid, reproductive, esophagus, brain, blood vessel); up	MTMR3<br>ASCC2<br>THOC5<br>SF3A1<br>NEFH<br>SEC14L3<br>DUSP18<br>GATSL3	no
**Necrosis of pulp or apical periodontitis**						
**2**	rs36044739	LRP1B	intron	no	no	no
**3**	rs62253088	CADM2	intron	no	no	no
**4**	rs79409042	TBCK	3-prime UTR	no	TBCK	no
**5**	rs6596024	RAPGEF6	intron	no	SLC22A5<br>RAPGEF6<br>SLC22A4<br>ACSL6<br>CDC42SE2<br>LYRM7<br>AC034220.3<br>HINT1<br>AC116366.5	no
**6**	rs9274719	HLA-DQB1	upstream gene	HLA-DRB1 (multiple); down<br>HLA-DQA1 (multiple); down<br>HLA-DQB1 (multiple); down<br>HLA-DQA2 (multiple); up<br>HLA-DQB2 (multiple); up<br>HLA-DQB1-AS1 (multiple); down	no	CCL19<br>HLA-A<br>HLA-DRA
**8**	rs35858015	MCPH1	regulatory region	no	no	no

**22**	rs9614155	HORMAD2	intron	AC003681.1 (MTMR3) (Multiple tissues); up	MTMR3<br>ASCC2<br>SF3A1<br>THOC5<br>GATSL3<br>NEFH<br>DUSP18	no

Expression quantitative trait loci (eQTLs) were analyzed with FIVEx database. The eQTL results were filtered for $p < 10^{-7}$. Protein quantitative loci (pQTLs) estimates for the FinnGen R12 were performed as a part of the FinnGen analysis pipeline. Association tests between SNPs and protein levels were run using PLINK. Proteins having a pQTL association with FDR <0.05 were considered statistically significant.

Supplementary Table 4. Gene Ontology of the protein-coding gene families for the endodontic phenotypes.

		Pulpal and apical diseases		Pulpitis		Necrosis of pulp or apical periodontitis	
**GO**	**+/-**	**Fold**	***P*-value**	**Fold**	***P*-value**	**Fold**	***P*-value**
**Biological process**							
Antigen processing and presentation of endogenous peptide antigen via MHC class II	+	>100	$2.49*10^{-6}$	>100	$1.79*10^{-6}$	>100	$1.19*10^{-6}$
Antigen processing and presentation of exogenous peptide antigen via MHC class II	+	>100	$3.53*10^{-14}$	>100	$9.83*10^{-15}$	>100	$6.92*10^{-13}$
Myeloid dendritic cell antigen processing and presentation	+	>100	$7.44*10^{-6}$	>100	$5.35*10^{-6}$	>100	$3.58*10^{-6}$
Peptide antigen assembly with MHC class II protein complex	+	>100	$1.56*10^{-16}$	>100	$4.34*10^{-17}$	>100	$7.61*10^{-15}$
Carnitine transmembrane transport	+	<0.01	>0.99	<0.01	>0.99	>100	$3.33*10^{-5}$
Positive regulation of CD4-positive, CD25-positive, alpha-beta regulatory T-cell differentiation	+	>100	$5.21*10^{-5}$	>100	$3.73*10^{-5}$	>100	$2.50*10^{-5}$
Positive regulation of memory T-cell differentiation	+	>100	$8.91*10^{-5}$	>100	$6.39*10^{-5}$	>100	$4.28*10^{-5}$
Positive regulation of antigen processing and presentation	+	>100	$1.11*10^{-4}$	<0.01	>0.99	89.48	$1.11*10^{-2}$
Positive regulation of memory T-cell mediated cytotoxicity	+	62.4	$4.79*10^{-7}$	55.16	$2.67*10^{-5}$	67.15	$1.45*10^{-4}$
Regulation of T-helper cell differentiation	+	38.18	$6.58*10^{-5}$	30.0	$2.02*10^{-3}$	54.78	$2.17*10^{-5}$
Detection of bacterium	+	38.98	$2.54*10^{-2}$	45.94	$2.16*10^{-2}$	>100	$1.42*10^{-4}$
Humoral immune response	+	9.20	$8.91*10^{-4}$	5.42	$5.22*10^{-2}$	13.21	$2.14*10^{-4}$
Adaptive immune response	+	9.13	$7.04*10^{-8}$	8.61	$2.44*10^{-6}$	9.17	$6.63*10^{-6}$
**Molecular function**							
MHC class II receptor activity	+	>100	$2.20*10^{-15}$	>100	$7.48*10^{-16}$	>100	$2.00*10^{-16}$
Carnitine transmembrane transporter activity	+	<0.01	>0.99	<0.01	>0.99	>100	$2.50*10^{-5}$
MHC class II protein complex binding	+	>100	$1.20*10^{-14}$	>100	$3.33*10^{-15}$	>100	$2.79*10^{-13}$
Natural killer cell lectin-like receptor binding	+	>100	$6.94*10^{-5}$	91.87	$1.08*10^{-2}$	<0.01	>0.99
Peptide antigen binding	+	97.01	$6.00*10^{-13}$	>100	$1.68*10^{-13}$	>100	$3.50*10^{-14}$
**Cellular component**							
Autolysosome membrane	+	>100	$5.21*10^{-5}$	>100	$3.73*10^{-5}$	>100	$2.50*10^{-5}$
MHC class II protein complex	+	>100	$2.65*10^{-16}$	>100	$7.36*10^{-17}$	>100	$1.18*10^{-14}$
Lumenal side of endoplasmic reticulum membrane	+	>100	$8.99*10^{-14}$	>100	$2.51*10^{-14}$	>100	$5.22*10^{-15}$
ER to Golgi transport vesicle membrane	+	69.29	$7.17*10^{-12}$	81.67	$2.01*10^{-12}$	99.42	$4.21*10^{-13}$
Clathrin-coated endocytic vesicle membrane	+	57.44	$2.79*10^{-11}$	67.70	$7.85*10^{-12}$	70.64	$1.99*10^{-10}$
Trans-Golgi network membrane	+	39.33	$4.17*10^{-10}$	46.35	$1.18*10^{-10}$	48.37	$2.01*10^{-9}$

Late endosome membrane	+	24.39	$1.18*10^{-8}$	28.74	$3.40*10^{-9}$	29.99	$3.55*10^{-8}$
Lysosomal membrane	+	9.49	$6.88*10^{-6}$	12.17	$1.32*10^{-6}$	12.70	$5.86*10^{-8}$
Golgi membrane	+	7.12	$1.14*10^{-5}$	8.39	$2.96*10^{-6}$	8.94	$7.86*10^{-6}$

Genes showing significant effects in eQTL and pQTL analyses were combined to Gene Ontology (GO) knowledgebase analyses. GO enrichment analysis was performed for biological processes, molecular functions, and cellular components. Results are filtered for False Discovery Rate (FDR) < 0.05 for at least one phenotype. Non-adjusted p-values are shown.

Supplementary Table 5. List of phenotypes included in the endpoint autoimmune diseases.

Endpoint name
rheumatoid arthritis
relapsing polychondritis
systemic lupus erythematosus
Sjögren syndrome
systemic scleroderma
dermatomyositis
Wegener granulomatosis
microscopic polyangiitis
Churg-Strauss syndrome
Henoch-Schoenlein purpura
rheumatic fever including heart disease
hypothyroidism, strict autoimmune
multiple sclerosis
type 1 diabetes
acute disseminated encephalomyelitis
other acute disseminated demyelination
narcolepsy and cataplexy
autoimmune hyperthyroidism
autoimmune thyroid disease
autoimmune polyglandular failure
autoimmune hepatitis
drug-induced autoimmune haemolytic anaemia
other autoimmune haemolytic anaemias
myasthenia gravis
other demyelinating diseases of the central nervous system
disorders of myoneural junction and muscle in diseases classified elsewhere
coeliac disease
primary biliary cirrhosis
Guillain-Barre syndrome
inflammatory bowel disease
psoriasis
vitiligo
alopecia areata
idiopathic thrombocytopenic purpura
Henoch-Schönlein purpura nephritis
adrenocortical insufficiency
hypersensitivity vasculitis
IgA nephropathy
vitamin B12 deficiency anaemia
anterior Iridocyclitis
graves ophthalmopathy, strict
Behçet disease
bullous pemphigoid
dermatitis herpetiformis
mixed connective tissue disease

Supplementary Table 6. Replication of the associations between endodontic phenotypes and HLA alleles in FinnGen-replication.

		FinnGen - replication					
		**Pulpal and apical diseases**		**Pulpitis**		**Necrosis of pulp or apical periodontitis**	
**HLA**	HLA allele	Beta (SE)	*P*-value	Beta (SE)	*P*-value	Beta (SE)	*P*-value
**A**	02:01	0.029 (0.031)	0.36	0.0247 (0.045)	0.55	0.022 (0.035)	0.52
	03:01	0.052 (0.034)	0.12	0.030 (0.048)	0.54	0.057 (0.037)	0.13
	32:01	**-0.254 (0.082)**	**0.002**[1]	**-0.318 (0.119)**	**0.006**[1]	**-0.172 (0.085)**	**0.04**[1]
**C**	01:02	-0.073 (0.056)	0.19	-0.076 (0.079)	0.34	-0.079 (0.061)	0.20
**DRB4**	01:03	**0.073 (0.033)**	**0.03**[1]	0.048 (0.047)	0.31	0.055 (0.036)	0.13
**DRB1**	01:01	-0.008 (0.035)	0.81	0.031 (0.050)	0.54	-0.016 (0.038)	0.67
	04:01	0.077 (0.050)	0.13	0.063 (0.072)	0.39	0.062 (0.056)	0.27
**DQA1**	01:01	-0.006 (0.034)	0.87	0.037 (0.050)	0.46	-0.016 (0.038)	0.67
	03:01	**0.100 (0.044)**	**0.02**[1]	**0.147 (0.064)**	**0.02**[1]	0.058 (0.049)	0.24
	03:03	0.008 (0.119)	0.94	-0.128 (0.173)	0.46	0.039 (0.131)	0.76
	05:05	-0.015 (0.060)	0.80	-0.097 (0.086)	0.26	0.001 (0.066)	0.99
**DQB1**	03:01	-0.019 (0.046)	0.68	-0.134 (0.066)	0.04[1]	0.012 (0.050)	0.81
	03:02	**0.088 (0.041)**	**0.03**[1]	**0.130 (0.059)**	**0.03**[1]	0.052 (0.046)	0.26
	05:01	-0.007 (0.033)	0.83	0.028 (0.048)	0.56	-0.021 (0.037)	0.57

Additive models adjusted for age, sex, and genetic principal components 1-10. Significant associations with the same direction as in the discovery cohort are bolded. $P < 0.05$ was considered significant in the replication analyses. Statistically significant *P*-values and associations with same effect direction as in the discovery analysis are bolded.

[1] Significant also in a model adjusted for age, sex, autoimmune diseases, and genetic principal components 1-10

Supplementary Table 7. Replication of the associations of KIR gene contents with endodontic phenotypes in FinnGen-replication.

		FinnGen-replication						
			**Pulpal and apical diseases**		**Pulpitis**		**Necrosis of pulp or apical periodontitis**	
		Frequency	Beta (SE)	*P*-value	Beta (SE)	*P*-value	Beta (SE)	*P*-value
**Inhibiting**	**2DL5**	0.74	-0.028 (0.039)	NS	-0.010 (0.056)	NS	-0.034 (0.043)	NS
**Activating**	**3DS1**	0.78	0.042 (0.040)	NS	0.040 (0.056)	NS	0.032 (0.043)	NS
	**2DS5**	0.82	0.035 (0.041)	NS	0.039 (0.058)	NS	0.026 (0.045)	NS

Additive model adjusted for age, sex, and genetic principal components 1-10. $P < 0.05$ was considered significant in the replication analyses. NS, not significant. SE, standard error.

Supplementary Table 8. Significant association of the top SNPs with other phenotypes in FinnGen.

Chr	SNP	Phenotype	*P*-value
**1**	rs2997084	Current smoker	$1.3*10^{-9}$
		Weight, inverse-rank normalized	$4.8*10^{-8}$
		Medical abortion	$7.9*10^{-8}$
		Substance abuse	$1.1*10^{-6}$
		Alcohol use disorder, ICD-based	$2.3*10^{-6}$
		Body-mass index, inverse-rank normalized	$4.7*10^{-6}$
		Paracetamol of NSAID medication	$9.4*10^{-5}$
**2**	rs143351662	NS	
**2**	rs36044739	Pregnancy with abortive outcome	$5.7*10^{-5}$
		Paracetamol of NSAID medication	$7.2*10^{-5}$
**3**	rs62253088	Weight, inverse-rank normalized	$1.1*10^{-7}$
		Glaucoma	$1.8*10^{-7}$
		Arthrosis	$4.8*10^{-7}$
		Body-mass index, inverse rank normalized	$7.7*10^{-6}$
		Gonarthrosis	$1.6*10^{-5}$
		Arthropathies	$3.0*10^{-5}$
		Height, inverse-rank normalized	$6.8*10^{-5}$
		Other localized connective tissue disorders	$8.8*10^{-5}$
**4**	rs79409042	Height, inverse-rank normalized	$4.4*10^{-6}$
**5**	rs6596024	Atopic dermatitis	$9.2*10^{-17}$
		Varicose veins	$7.3*10^{-15}$
		Dermatitis and eczema	$1.5*10^{-14}$
		Nasal polyp	$1.2*10^{-12}$
		Chronic lower respiratory diseases	$1.7*10^{-8}$
		Asthma/COPD	$1.8*10^{-8}$
		Dermatitis	$2.6*10^{-6}$
		Coronary revascularization	$3.1*10^{-6}$
		Weight, inverse-rank normalized	$4.6*10^{-6}$
		Malignant neoplasm of breast	$9.1*10^{-6}$
		Gonarthrosis	$1.3*10^{-5}$
		Allergic contact dermatitis	$1.8*10^{-5}$
		Angina pectoris	$3.3*10^{-5}$
		Wet age-related macular degeneration	$3.9*10^{-5}$
		Ischaemic heart disease, wide definition	$6.0*10^{-5}$
		Polymyalgia rheumatica	$7.3*10^{-5}$
**6**	rs9449423	NS	
**6**	rs9270664, rs9274724, rs9270911	Coeliac disease	$< 5*10^{-324}$
		Type 1 diabetes	$< 5*10^{-324}$
		Autoimmune diseases	$9.1*10^{-225}$
		Hypothyroidism, strict autoimmune	$6.8*10^{-162}$
		Diabetic retinopathy	$1.5*10^{-105}$
		Diseases of the digestive system	$5.2*10^{-76}$
		Other retinal disorders	$2.9*10^{-68}$
		Diabetic maculopathy	$3.6*10^{-57}$
		Disorders of choroid and retina	$2.5*10^{-48}$
		MS-disease / Multiple Sclerosis	$2.5*10^{-42}$
		Seropositive rheumatoid arthritis	$1.2*10^{-39}$

		Dermatitis herpetiformis	$6.5*10^{-38}$
		Bullous disorders	$6.4*10^{-34}$
		Lichen ruber planus	$1.2*10^{-32}$
		Chronic lower respiratory diseases	$1.1*10^{-31}$
		Diabetic nephropathy	$1.1*10^{-30}$
		Asthma/COPD	$9.6*10^{-29}$
		Type 2 diabetes	$1.1*10^{-26}$
		Ankylosing spondylitis	$1.4*10^{-18}$
		Iridocyclitis	$4.3*10^{-18}$
		Sarcoidosis	$5.8*10^{-15}$
		Anaemias	$4.3*10^{-13}$
		And numerous other diseases of autoimmune character	
**8**	rs35858015	Viral warts	$2.0*10^{-5}$
		Nail dystrophy	$5.9*10^{-5}$
**9**	rs1417738	Height, inverse-rank normalized	$3.7*10^{-5}$
		Diseases of vocal cords and larynx	$7.3*10^{-5}$
		Spinal instabilities	$9.2*10^{-5}$
**14**	rs1245193	NS	
**17**	rs1292071	Iron deficiency anaemia	$4.1*10^{-8}$
		Autoimmune diseases excluding thyroid diseases	$3.3*10^{-6}$
		Anaemias	$2.6*10^{-5}$
		Other and unspecified nerve root and plexus disorders, also in other diseases	$2.6*10^{-5}$
		Autoimmune diseases	$3.0*10^{-5}$
		COPD	$3.1*10^{-5}$
		Neuralgia and neuritis	$3.4*10^{-5}$
		Other ulcerative colitis	$6.8*10^{-5}$
**18**	rs8088342	Acidosis	$2.1*10^{-5}$
**22**	rs9614152, rs9614155, rs9614155	Chronic diseases of tonsils and adenoids	$1.6*10^{-22}$
		Tonsillectomy and adenotomy	$6.5*10^{-16}$
		Other diseases of upper respiratory tract	$6.8*10^{-10}$
		Disorders of the thyroid gland	$3.0*10^{-6}$
		Antihypertensive medication	$1.7*10^{-5}$
		Height, inverse-rank normalized	$3.9*10^{-5}$
		Erosion of teeth	$6.1*10^{-5}$

NS, no significant associations with any phenotype. *P*-values $<1.0*10^{-4}$ were considered statistically significant.

Supplementary Table 9. Observed heritability estimates of the FinnGen phenotypes.

Phenotype	H^2	SE
BMI	0.187	0.008
Current smoking status	0.104	0.004
Caries	0.091	0.007
Atrial fibrillation and flutter	0.080	0.008
Hypertension, essential	0.078	0.003
Statin medication	0.074	0.007
Type 2 diabetes	0.069	0.003
Autoimmune diseases[1]	0.050	0.007
Asthma/COPD	0.049	0.003
Pain	0.048	0.0019
Cardiovascular diseases	0.047	0.002
Coronary revascularization	0.044	0.003
Coronary atherosclerosis	0.043	0.003
Disorders of the thyroid gland	0.043	0.005
Chronic diseases of tonsils and adenoids	0.040	0.003
Ischaemic heart disease	0.040	0.002
Angina pectoris	0.037	0.002
Coeliac disease	0.036	0.027
Major coronary heart disease event	0.035	0.002
Myocardial infarction, strict	0.032	0.002
Rheumatoid arthritis	0.030	0.007
Migraine	0.029	0.002
Disorders of lipoprotein metabolism and other lipidaemias	0.025	0.002
Type 1 diabetes	0.024	0.014
Stroke	0.022	0.002
Pulpitis	0.021	0.001
Age-related macular degeneration	0.018	0.006
Unstable angina pectoris	0.018	0.002
Endodontic infections	0.018	0.001
Necrosis of pulp or apical periodontitis	0.017	0.001
Stroke, excluding SAH	0.011	0.001

We estimated SNP-based observed scale heritability (H^2) by performing linkage disequilibrium score regression. SE, standard error.

[1] A composite phenotype Autoimmune diseases specified in Table S5.

Supplementary Table 10. Genetic correlations between endodontic phenotypes and selected FinnGen phenotypes.

	Pulpal and apical diseases			Pulpitis			Necrosis of pulp or apical periodontitis		
**Phenotype**	r_g	**SE**	***P*-value**	r_g	**SE**	***P*-value**	r_g	**SE**	***P*-value**
Pain	0.60	0.03	**$1.5*10^{-6}$**	0.63	0.03	**$5.9*10^{-120}$**	0.57	0.03	**$1.2*10^{-70}$**
Caries	0.54	0.04	**$8.5*10^{-34}$**	0.57	0.05	**$5.5*10^{-31}$**	0.55	0.05	**$7.05*10^{-31}$**
Current smoking status	0.41	0.03	**$2.7*10^{-38}$**	0.44	0.03	**$1.3*10^{-46}$**	0.42	0.03	**$5.0*10^{-34}$**
BMI	0.41	0.02	**$4.6*10^{-67}$**	0.40	0.02	**$5.9*10^{-60}$**	0.43	0.03	**$2.9*10^{-60}$**
Cardiovascular diseases	0.45	0.03	**$4.6*10^{-67}$**	0.39	0.03	**$1.1*10^{-47}$**	0.43	0.03	**$1.2*10^{-46}$**
Migraine	0.31	0.04	**$3.1*10^{-14}$**	0.35	0.04	**$4.5*10^{-17}$**	0.26	0.04	**$8.2*10^{-10}$**
Stroke	0.38	0.05	**$2.2*10^{-17}$**	0.32	0.04	**$2.7*10^{-13}$**	0.39	0.05	**$4.2*10^{-16}$**
Disorders of lipoprotein metabolism and other lipidaemias	0.32	0.04	**$3.7*10^{-15}$**	0.31	0.04	**$6.3*10^{-14}$**	0.33	0.04	**$1.9*10^{-13}$**
Hypertension, essential	0.34	0.03	**$9.6*10^{-38}$**	0.31	0.03	**$1.1*10^{-33}$**	0.33	0.03	**$2.1*10^{-27}$**
Unstable angina pectoris	0.32	0.04	**$7.3*10^{-14}$**	0.30	0.04	**$1.1*10^{-13}$**	0.31	0.05	**$2.7*10^{-11}$**
Angina pectoris	0.31	0.03	**$6.2*10^{-19}$**	0.30	0.03	**$1.5*10^{-18}$**	0.31	0.04	**$5.9*10^{-17}$**
Type 2 diabetes	0.29	0.03	**$3.9*10^{-25}$**	0.29	0.03	**$7.2*10^{-23}$**	0.30	0.03	**$2.0*10^{-23}$**
Ischaemic heart disease	0.32	0.03	**$3.2*10^{-24}$**	0.28	0.03	**2.6*10-^{20}**	0.33	0.03	**$3.3*10^{-22}$**
Rheumatoid arthritis	0.32	0.04	**$2.1*10^{-14}$**	0.28	0.04	**$2.1*10^{-11}$**	0.32	0.05	**$1.4*10^{-11}$**
Chronic diseases of tonsils and adenoids	0.30	0.04	**$2.4*10^{-11}$**	0.27	0.04	**$1.7*10^{-10}$**	0.29	0.05	**$4.6*10^{-10}$**
Asthma/COPD	0.31	0.03	**$2.9*10^{-22}$**	0.27	0.03	**$4.7*10^{-15}$**	0.32	0.04	**$1.5*10^{-19}$**
Coronary atherosclerosis	0.29	0.03	**$3.5*10^{-20}$**	0.25	0.03	**$5.0*10^{-17}$**	0.31	0.03	**$7.2*10^{-19}$**
Autoimmune diseases	0.25	0.03	**$3.0*10^{-17}$**	0.24	0.03	**$3.5*10^{-16}$**	0.25	0.03	**$1.2*10^{-14}$**
Stroke, excluding SAH	0.32	0.05	**$2.7*10^{-9}$**	0.23	0.05	**$1.4*10^{-05}$**	0.33	0.06	**$3.6*10^{-09}$**
Major coronary heart disease event	0.25	0.03	**$8.1*10^{-13}$**	0.23	0.03	**$7.1*10^{-13}$**	0.26	0.04	**$2.1*10^{-11}$**
Atrial fibrillation and flutter	0.29	0.03	**$6.0*10^{-22}$**	0.22	0.03	**$2.9*10^{-15}$**	0.27	0.03	**$1.8*10^{-16}$**
Myocardial infarction, strict	0.23	0.04	**$1.5*10^{-9}$**	0.21	0.04	**$1.6*10^{-09}$**	0.24	0.04	**$1.5*10^{-09}$**
Statin medication	0.22	0.03	**$2.6*10^{-13}$**	0.20	0.03	**$1.0*10^{-11}$**	0.23	0.03	**$3.5*10^{-13}$**
Coronary revascularization	0.20	0.03	**$2.0*10^{-9}$**	0.18	0.03	**$5.6*10^{-09}$**	0.21	0.04	**$1.3*10^{-08}$**
Disorders of the thyroid gland	0.17	0.03	**$2.0*10^{-7}$**	0.16	0.03	**$5.6*10^{-08}$**	0.16	0.04	**$3.1*10^{-06}$**
Age-related macular degeneration	0.13	0.06	0.04	0.14	0.06	0.01	0.13	0.07	0.061
Type 1 diabetes	0.04	0.04	0.32	0.08	0.04	0.03	0.03	0.05	0.55
Coeliac disease	-0.04	0.04	0.32	0.00	0.06	0.98	-0.05	0.05	0.33

Genetic correlations (r_g) were estimated using linkage disequilibrium score regression. The threshold for statistical significance was $P < 0.00056$. Statistically significant *P*-values are bolded. SE, standard error.
