# Supplementary material for: Genome-wide association study of pulpal and apical diseases
Source: Nat Commun. 2025 Jul 23;16:6774. doi: 10.1038/s41467-025-61721-1 (PMC12287303; doi:10.1038/s41467-025-61721-1)
Supplement: Supplementary file 2 — Description of Additional Supplementary Files [file 41467_2025_61721_MOESM2_ESM.pdf]

Description of Additional Supplementary Files

Supplementary Data 1: List of genome-wide significant SNPs for phenotypes Pulpal and apical diseases, Pulpitis, and Necrosis of pulp or apical periodontitis.

Supplementary Data 2: Associations between lead SNPs and three phenotypes adjusted for DMFS, and results from sex-specific analyses.

Supplementary Data 3: Associations between lead SNPs and dental caries diagnosis codes in FinnGen (ICD-10 K02 category and K02.1).

Supplementary Data 4: RNAseq expression of genes located in the significant loci in human dental pulp and in developing mouse tooth, and their differential expression in pulpitis.

Supplementary Data 5: Regulatory significance scores of most credible variants.

Supplementary Data 6: List of FinnGen Consortium members, their affiliations, and contributions to the FinnGen project.
