# Supplementary material for: Genome-wide association study of pulpal and apical diseases
Source: Nat Commun. 2025 Jul 23;16:6774. doi: 10.1038/s41467-025-61721-1 (PMC12287303; doi:10.1038/s41467-025-61721-1)
Supplement: Supplementary file 4 — Reporting Summary [file 41467_2025_61721_MOESM4_ESM.pdf]

nature portfolio

Corresponding author(s): Aino Salminen

Last updated by author(s): May 22, 2025

Reporting Summary

Nature Portfolio wishes to improve the reproducibility of the work that we publish. This form provides structure for consistency and transparency in reporting. For further information on Nature Portfolio policies, see our Editorial Policies and the Editorial Policy Checklist.

Statistics

For all statistical analyses, confirm that the following items are present in the figure legend, table legend, main text, or Methods section.

n/a	Confirmed	
☐	☒	The exact sample size (*n*) for each experimental group/condition, given as a discrete number and unit of measurement
☒	☐	A statement on whether measurements were taken from distinct samples or whether the same sample was measured repeatedly
☐	☒	The statistical test(s) used AND whether they are one- or two-sided<br>*Only common tests should be described solely by name; describe more complex techniques in the Methods section.*
☐	☒	A description of all covariates tested
☐	☒	A description of any assumptions or corrections, such as tests of normality and adjustment for multiple comparisons
☐	☒	A full description of the statistical parameters including central tendency (e.g. means) or other basic estimates (e.g. regression coefficient) AND variation (e.g. standard deviation) or associated estimates of uncertainty (e.g. confidence intervals)
☐	☒	For null hypothesis testing, the test statistic (e.g. *F*, *t*, *r*) with confidence intervals, effect sizes, degrees of freedom and *P* value noted<br>*Give P values as exact values whenever suitable.*
☒	☐	For Bayesian analysis, information on the choice of priors and Markov chain Monte Carlo settings
☒	☐	For hierarchical and complex designs, identification of the appropriate level for tests and full reporting of outcomes
☒	☐	Estimates of effect sizes (e.g. Cohen's *d*, Pearson's *r*), indicating how they were calculated

Our web collection on statistics for biologists contains articles on many of the points above.

Software and code

Policy information about availability of computer code

Data collection	A detailed description of data production is available at https://finngen.gitbook.io/documentation.
Data analysis	Association analyses were performed with REGENIE (v2.2.4): https://rgcgithub.github.io/regenie/, PLINK (v2.0): https://www.cog-genomics.org/plink/2.0/, and SNPtest (v2.5.4): https://www.chg.ox.ac.uk/~gav/snptest/.<br><br>Finemapping was performed with SuSiE: https://github.com/stephenslab/susieR. Details on finemapping are available at https://github.com/FINNGEN/finemapping-pipeline.<br><br>Genetic correlation were analyzed with LDSC (v1.0.1): https://github.com/bulik/ldsc.

For manuscripts utilizing custom algorithms or software that are central to the research but not yet described in published literature, software must be made available to editors and reviewers. We strongly encourage code deposition in a community repository (e.g. GitHub). See the Nature Portfolio guidelines for submitting code & software for further information.

Data

Policy information about availability of data

All manuscripts must include a data availability statement. This statement should provide the following information, where applicable:

- Accession codes, unique identifiers, or web links for publicly available datasets
- A description of any restrictions on data availability
- For clinical datasets or third party data, please ensure that the statement adheres to our policy

The GWAS summary statistics generated in this study are available in the FinnGen public cloud bucket (https://storage.googleapis.com/fg-publication-green-public/F_2023_050_20250522/summary_statistics_pulpal_and_apical_diseases.zip). The individual-level data from FinnGen are available under restricted access due to the sensitive nature of the genotype and phenotype information. Individual-level genotypes and register data from FinnGen participants can be applied via the Fingenious® services (https://site.fingenious.fi/en/) hosted by the Finnish Biobank Cooperative FinBB (https://finbb.fi/en/). Finnish Health register data can be applied by approved researchers via Finnish Data Authority Findata (https://findata.fi/en/data/).

Research involving human participants, their data, or biological material

Policy information about studies with human participants or human data. See also policy information about sex, gender (identity/presentation), and sexual orientation and race, ethnicity and racism.

Reporting on sex and gender	Sex of the participants was determined by genotyping and from the health registers. Individuals with ambiguous sex were excluded from the analyses. Results from sex-specific GWAS analyses are available in Supplementary Data 2.
Reporting on race, ethnicity, or other socially relevant groupings	Ancestry was defined based on genetic data. Other information on ethnicity was not collected. In FinnGen, individuals with non-Finnish ancestry were removed from the analyses.
Population characteristics	FinnGen Release 12 (September 2023) included data from 520,210 participants. The number of participants with pulpal or apical diseases was 132,124 and their mean age was 60.9 years.
Recruitment	FinnGen study is a nationwide collection of genetic samples combining genetic data with health register data. Participants include legacy samples from previous sample collecions and recent biobank samples recruited at university hospitals across Finland and from the Finnish Blood Service. Details on recruitment are available at https://www.finngen.fi/en/node/1985.
Ethics oversight	All studies were done in accordance with the Declaration of Helsinki. Based on the Finnish biobank act, participants entered the FinnGen study by signing an informed consent for biobank research. The Coordinating Ethics Committee of the Hospital District of Helsinki and Uusimaa approved the FinnGen study protocol Nr HUS/990/2017. The activities of the EstBB are regulated by the Human Genes Research Act. Individual level data analysis in EstBB was carried out under ethical approval 1.1-12/624 from the Estonian Committee on Bioethics and Human Research (Estonian Ministry of Social Affairs), using data according to release application 6-7/GI/33501 from the Estonian Biobank. An informed consent was obtained from participants in the NFBC66/86 cohorts. The ethics committee of the Northern Ostrobothnia Hospital District approved the study plans.

Note that full information on the approval of the study protocol must also be provided in the manuscript.

Field-specific reporting

Please select the one below that is the best fit for your research. If you are not sure, read the appropriate sections before making your selection.

☒ Life sciences ☐ Behavioural & social sciences ☐ Ecological, evolutionary & environmental sciences

For a reference copy of the document with all sections, see nature.com/documents/nr-reporting-summary-flat.pdf

Life sciences study design

All studies must disclose on these points even when the disclosure is negative.

Sample size	We used the FinnGen data release 12, which included data from 520,210 participants. Using national health registers, we identified 132,124 cases with pulpal and apical diseases, 48,120 cases with pulpitis, and 103,832 cases with necrosis of pulp or apical periodontitis for the discovery analyses. The number of controls without diagnosis for pulpal and apical diseases was 353,106.
Data exclusions	In sample-wise quality control, individuals with ambiguous gender, high genotype missingness (>5%), excess heterozygosity (+-4SD), and non-Finnish ancestry were excluded. In variant-wise quality control, variants with high missingness (>2%), low HWE p-value (<10-6) and low minor allele count (MAC<3) were excluded.
Replication	Findings from the discovery analysis were replicated in three replication populations: FinnGen-replication, Estonian Biobank, and Northern Finland Birth Cohorts 1966 and 1986.
Randomization	This is not relevant in our study. Our study is a genome-wide association study using register-based data to define cases and controls.

Randomization	Randomization is not applicable in the methodology of this study.
Blinding	This is not relevant in our study. Our study is a genome-wide association study using register-based data to define cases and controls. Blinding is not applicable in the methodology of this study.

Reporting for specific materials, systems and methods

We require information from authors about some types of materials, experimental systems and methods used in many studies. Here, indicate whether each material, system or method listed is relevant to your study. If you are not sure if a list item applies to your research, read the appropriate section before selecting a response.

Materials & experimental systems

n/a	Involved in the study
☒	☐ Antibodies
☒	☐ Eukaryotic cell lines
☒	☐ Palaeontology and archaeology
☒	☐ Animals and other organisms
☒	☐ Clinical data
☒	☐ Dual use research of concern
☒	☐ Plants

Methods

n/a	Involved in the study
☒	☐ ChIP-seq
☒	☐ Flow cytometry
☒	☐ MRI-based neuroimaging

Plants

Seed stocks	*Report on the source of all seed stocks or other plant material used. If applicable, state the seed stock centre and catalogue number. If plant specimens were collected from the field, describe the collection location, date and sampling procedures.*
Novel plant genotypes	*Describe the methods by which all novel plant genotypes were produced. This includes those generated by transgenic approaches, gene editing, chemical/radiation-based mutagenesis and hybridization. For transgenic lines, describe the transformation method, the number of independent lines analyzed and the generation upon which experiments were performed. For gene-edited lines, describe the editor used, the endogenous sequence targeted for editing, the targeting guide RNA sequence (if applicable) and how the editor was applied.*
Authentication	*Describe any authentication procedures for each seed stock used or novel genotype generated. Describe any experiments used to assess the effect of a mutation and, where applicable, how potential secondary effects (e.g. second site T-DNA insertions, mosiacism, off-target gene editing) were examined.*
